# The Cytotoxic Properties of Extreme Fungi’s Bioactive Components—An Updated Metabolic and Omics Overview

**DOI:** 10.3390/life13081623

**Published:** 2023-07-25

**Authors:** Attila Kiss, Farhad Hariri Akbari, Andrey Marchev, Viktor Papp, Iman Mirmazloum

**Affiliations:** 1 Agro-Food Science Techtransfer and Innovation Centre, Faculty for Agro, Food and Environmental Science, Debrecen University, 4032 Debrecen, Hungary; attkiss@agr.unideb.hu; 2 Department of Biology, Biotechnical Faculty, University of Ljubljana, 1000 Ljubljana, Slovenia; hariri.a.farhad@gmail.com; 3 Laboratory of Metabolomics, Department of Biotechnology, The Stephan Angeloff Institute of Microbiology, Bulgarian Academy of Sciences, 4000 Plovdiv, Bulgaria; 4 Department of Botany, Hungarian University of Agriculture and Life Sciences, 1118 Budapest, Hungary; papp.viktor@uni-mate.hu; 5 Department of Plant Physiology and Plant Ecology, Institute of Agronomy, Hungarian University of Agriculture and Life Sciences, 1118 Budapest, Hungary

**Keywords:** extremophile, extremotolerance, antitumor, novel cancer treatments, secondary metabolites, multiomics

## Abstract

Fungi are the most diverse living organisms on planet Earth, where their ubiquitous presence in various ecosystems offers vast potential for the research and discovery of new, naturally occurring medicinal products. Concerning human health, cancer remains one of the leading causes of mortality. While extensive research is being conducted on treatments and their efficacy in various stages of cancer, finding cytotoxic drugs that target tumor cells with no/less toxicity toward normal tissue is a significant challenge. In addition, traditional cancer treatments continue to suffer from chemical resistance. Fortunately, the cytotoxic properties of several natural products derived from various microorganisms, including fungi, are now well-established. The current review aims to extract and consolidate the findings of various scientific studies that identified fungi-derived bioactive metabolites with antitumor (anticancer) properties. The antitumor secondary metabolites identified from extremophilic and extremotolerant fungi are grouped according to their biological activity and type. It became evident that the significance of these compounds, with their medicinal properties and their potential application in cancer treatment, is tremendous. Furthermore, the utilization of omics tools, analysis, and genome mining technology to identify the novel metabolites for targeted treatments is discussed. Through this review, we tried to accentuate the invaluable importance of fungi grown in extreme environments and the necessity of innovative research in discovering naturally occurring bioactive compounds for the development of novel cancer treatments.

## 1. Introduction

Cancer is a polygenic, multifactorial, and complex disease comprising up to 300 different types that are associated with various environmental factors and genetic disorders since the dawn of mankind [1,2,3]. Documented data indicate that cancer is the second leading cause of death after cardiovascular disease [4]. An estimated 9.6 million deaths related to cancer since 2018 were reported by a study on the global burden of disease [3,5].

Depending on the disease conditions, there are various prescribed treatments for cancer, including radiotherapy, chemotherapy, targeted surgery and immunotherapy, recent gene therapies, or a combination of the above approaches [6,7,8].

There are usually conjugated elements in cancer therapies that deliver drugs to specific cancer cells or peripheral tissues that prevent tumour cells from growing [9]. So far, several cytotoxic compounds have been identified with chemotherapeutic properties, of which most are non-tumour-cell-specific and cause toxicity to healthy tissue [10]. Moreover, the chemo-resistance phenomenon is still a weakness of these treatments [11]. Despite numerous advances in cancer treatment, the resistance of mammalian tumour cells to chemotherapy and their numerous side effects necessitates the discovery and introduction of newer alternatives or synergistic drugs [12].

In contrast, natural products derived from various microorganisms with a variety of medicinal properties such as targeted cytotoxic, antitumor, anti-inflammatory, and other activities are continuously being discovered [13]. Such secondary metabolites, also referred to as “specialized metabolites” in the recent literature, are organic compounds that, unlike primary metabolites, are not directly involved in the growth or reproduction of a living organism [14,15,16]. It is believed that these compounds are being utilized to protect the organisms from various biotic and abiotic stress factors [17,18,19].

Despite great progress in the synthesis of synthetic substances, novel metabolites produced in plants/fungi with medicinal properties often have complex biosynthetic pathways with complicated structures, which makes it difficult to synthesize them artificially on an industrial scale [19,20].

As a result, the most common accessibility options for such metabolites are natural resources and targeted extraction and purification [21]. 

Fungi are known to be the most diverse and abundant organisms on our planet [22]. Several fungal species are able to grow/thrive in extreme environments [23]. The presence of fungi in all kinds of ecosystems provides excellent opportunities for the research and discovery of new natural products [24,25]. Fungi-derived compounds are widely used in medical, biotechnological, and traditional bioremediation applications [26,27,28,29]. They have also been the subject of several studies to obtain new biomolecules and enzymes, serving various purposes such as biofuels, insecticides, pesticides, anthelmintic, and analgesics. Additionally, fungi’s metabolites are utilized in flavourings, cosmetics, and as supplements in single-cell proteins (SCPs) [30,31,32].

The profound value and importance of these compounds in the mentioned industries is completely consistent with the evolving desire of our society towards natural treatments. Due to this growing trend, it has become necessary to perform an extensive chemical discovery of these metabolites, with the aim of increasing our understanding of their biosynthetic origin and unlocking their enormous potential for diverse biological activities [33,34]. In the field of fungal discoveries, penicillin produced by *Penicillium notatum* has been considered a goldmine since 1928 [35]. Ascomycetes such as *Aspergillus, Penicillium,* and *Fuserium*, as well as imperfect fungi, are common producers of bioactive compounds, followed by the endophytic [35,36,37] and filamentous species, and *Alterneria, Trichoderma,* and *Phoma* [38]. However, there are still several metabolites of these microorganisms that are yet to be characterized [39]. 

The term “extremus” means extreme in Latin and Greek, and the word “philiā” conveys the meaning of love [40,41]. The extremus organisms have existed for many years and continue to grow under extreme conditions, making them excellent choices to be considered for evolutionary studies [42,43]. According to the concept derived from these terms, these organisms can grow in harsh physical and geochemical conditions that are intolerable to most organisms [44]. They can proliferate in salty, acidic, and alkaline conditions and solutions; in hot and icy recesses; in toxic wastes, heavy metals, and organic solvents; or in other extreme habitats which, as mentioned earlier, are considered unfavorable for most organisms [45,46].

These organisms are typically classified into three groups: I: extremophilic organisms, which require severe conditions to reproduce [46]; II: extremotolerant organisms, which grow optimally under “normal” conditions but can tolerate extreme amounts of one or more unfavourable physico-chemical and environmental parameters [47]; and finally, III: polyextremophilic or polyextremotolerant organisms, which adapt to or survive in areas where extremely unfavourable physico-chemical parameters are present [48,49]. In addition, they can be also classified based on the environments in which they can thrive, for instance, the organisms that grow at high temperatures are termed thermophilic and hyperthermophilic; the psychrophilics that can grow at low temperatures; or those who are adapted to acidic or basic pH environments and are referred to as acidophilic or alkaliphilic. The species that grow in high-pressure conditions, such as in the deep sea or underground, are called piezophilic or barophilic, and finally, there are organisms that require NaCl to grow or survive in environments with high salt concentrations and low water activity_(aw)_ that are generally called halophilic [50,51].

Incidentally, the molecular perception and mechanism for survival in such extreme ecosystems has been studied [23]. Apparently, structural changes and biologically active metabolites that are catalytically active under these conditions seem to play an essential role in tolerance and adaptability [52,53]. Biodiversity in the microbial community produces a wide range of secondary metabolites that are limited by many factors and change under stress condition [17,54]. This means that under high stress condition, biodiversity tends to be lower whereas, species diversity can increase when stress level decrease [55,56]. Now, we know that increased levels of stress can induce genetic mutations in all living organisms, and sometimes, the mutations and survival are considered as an alternative method to adapt, proliferate, and grow in extreme conditions, with consequent production of important metabolites with medicinal properties [57,58,59].

Natural products with medicinal or anticancer properties are called bioactive molecules that are produced by living organisms [60]. Although the majority of natural anticancer products are derived from plants, microorganisms, especially fungi, can also be considered as excellent sources of these ingredients, with even more attractive properties [61,62]. They can be manipulated easily and screened biologically and physiologically to discover novel natural products with extraordinary diversity among living organisms [63]. The metabolites produced by extremophiles and extremotolerant fungi are very valuable to produce drugs against cancer cells [64]. In addition, fungi produce other bioactive metabolites such as mycotoxins, antimycotics [65,66,67], immunosuppressants [68], antifungals [69], antibacterials [70], and antivirals [71]. Even though intensive research has been conducted on fungi-derived natural products and their medicinal properties for more than a century, there are still several fungal metabolites with unknown biological activities that can be considered as new sources of drugs [37]. 

The following sections briefly review some of the major bioactive antitumor (anticancer) metabolites isolated from extremophilic, extremotolerant, and polyextremophilic (or tolerant) fungi reported in the published literature from 1984 to 2023; the reported effective doses, the types of target cells and the omics approaches used for their identification are also addressed.

## 2. Fungi of Extreme Temperature Conditions

Temperature is one of the important factors that cause selective pressure on microorganisms like fungi [72] and may induce the integration of cellular components, which leads to structural and functional alterations of biological molecules [73,74]. Psychrophiles are one type of extremophiles which grow and live in temperatures lower than 15 degrees Celsius [75,76], while psychrotolerants proliferate optimally and grow at 15–30 °C [77,78]. Several species of micro-fungi have been reported from the Antarctic region that can survive the subzero temperatures [79]. On the other hand, thermophilic fungi are extremophiles that can proliferate at approximately 45 °C and can tolerate the higher temperatures of up to 61 °C [74,80]. The unique special compounds of these fungi (e.g., heat-resistant enzymes) can be of high interest for modern biotechnology [81,82]. The biosynthetic pathways, structures, and applications of these bioactive compounds are yet to be fully characterized [82,83,84].

Psychrophilic or psychrotolerant fungi produce certain metabolites at low temperatures that are perhaps essential for their survival in harsh environments such as the deep sea of oceans with cold water, high pressure, and nutrient deficiencies [85,86]. These fungi are classified as polyextremophile or tolerant to multiple environmental stresses [87]. 

So far, only a limited number of studies have been conducted to identify biologically active compounds from this group of extreme fungi [88]. As instances of these calls of compounds with cytotoxic activity against tumour cell lines, a novel cyclic nitropeptide named Psychrophilin A–D from *Penicillium algidus* (a psychrophilic fungi) was isolated from soil specimens in Greenland [89]. Psychrophilin A–D showed moderate cytotoxic activity with an effective dose (ID_50_) value of 10.1 μg/mL against P388 murine leukemia cell lines [89]. Piperazine-type compounds, including Oidioperazines A–D and Chetracins B–D produced by the fungus *Oidiodendron truncatum* GW3-13, were also isolated from soil samples in the Antarctic station near the Great Wall of China [77]. The cytotoxic activity of these piperazine-type compounds against A2780, Bel-7402, HCT-8, BGC-823, and A549 cell lines was documented [42,65].

Thermophilic or thermotolerant fungi are microorganisms that live in hot springs with temperatures between 45 °C and 65 °C [74]. A review of articles on the characterization of fungi in such a harsh environment revealed the incompleteness in the structural clarification, identification, and biological activities of their metabolites. *Aspergillus terreus* is one thermophilic fungus that produces lovastatin, which has a selective prohibitory effect on primary AML cell growth up to 75–95%. Moreover, the detracted proliferation by lovastatin have been reported with moderate inhibitory effect and induced apoptosis on four lung cancer cell lines and in ten ovarian cancer cell lines, respectively [31,46]. In addition, it has been recently found that lovastatin with IC_50_ values of 0.6, 0.7, and 1.1 μg/mL inhibited the liver cancer HepG_2_, cervical cancer HeLa, and breast cancer MCF-7 cell lines, respectively [37]. 

Simvastatin is another metabolite that was extracted from *A. terreus* with prohibitive effect on the growth of three melanoma, two lung, and four breast cancer cell lines and with an apoptosis effect of reducing tumour growth in hepatic cancer cells with IC_50_ values between 0.8 and 5.4 μM, making it an anticancer drug candidate for different clinical experiments [37]. 

Asperlin and brefeldin A are small polyketides produced by *Aspergillus terreus* (*JAS-2*) whose anticancer activities have been demonstrated for decades after their discovery [37]. Terrein, a compound isolated also from *A. terreus*, is a small antifungal agent known since 1935 and widely studied for its anticancer properties for almost 80 years. Terrein with an IC_50_ value of 1.1 nM was reported to cause apoptosis in breast cancer (MCF-7 cell line); in addition, its activity against liver cancer cell lines of HepG_2_ (IC_50_ 66.8 μM) and pancreatic PANC-1 (IC_50_ 9.8 μM) was also reported [37]. It is noteworthy to mention that terrein’s activity against the mentioned cancer cell line was 100 times stronger than Taxol, which is extracted from *Taxus baccata* [37]. 

Fumagillin is another metabolite first isolated from the liquid culture of *Aspergillus fumigatus* strain H-3.77 and later found in other species of Aspergillus such as *A. flavus* and *A. parasiticus*. One of the chlorinated derivatives of fumagillin was extracted from a marine-inhabiting Penicillium strain with potent inhibitory activity against osteosarcoma cell lines [90,91,92]. TNP-470, PPI-2458, and CKD-732 are synthetically derived fumagillin analogs that have been tested in human cancer clinical experiments. These molecules interact with the enzyme methionine aminopeptidase type 2 that cleaves the N-terminal methioninyl residue of newly synthesized proteins, interrupting the tumour vessels [91]. 

Chromatographic separation and molecular identification of the obtained fragments of *Malbranchea sulfurea* metabolites revealed six light-sensitive polyketides called malbran pyrroles A–F with cytotoxic activities against PANC-1, HepG_2_, and MCF-7 cancer cell lines, with their IC_50_ values in the range from 3 to 11 μM 42 [42,51]. More metabolites of this group of fungi and their biological activities are listed in Table 1, with their half inhibitory concentrations (IC50), specifically on human cancer cell lines, unless otherwise, stated. These IC50 values serve as critical reference points in determining the potency and efficacy of these agents against various cancers. 

## 3. Piezophilic Fungi

Piezophiles are organisms that proliferate optimally under high pressure (≤100 MPa), while piezo-tolerants can grow optimally at pressures above 0.1 MPa [42]. Piezophilic fungi adapt and grow in deep biospheres of the sea and oceans [105]. In research on natural medicinal products, the marine Acremonium fungi gained more attention for their antimicrobial cephalosporins compared to their less explored terrestrial counterparts [42,106]. Piezophiles and piezo-tolerants of extreme environments deserve more exploration due to their ability to produce novel biologically active compounds with potential therapeutic properties [107]. However, it should be emphasized that the research on these fungi and their secondary metabolites is very confined because of the sampling challenges and the limitation of adequate culture simulation in the laboratory. However, the advanced execution of deep-sea sampling and drilling methods, as well as omics technologies and culture-dependent methods, are being used as part of current approach to identify cytotoxic compounds and metabolites from the marine environment [30,68,108]. A novel hydroxyphenylacetic acid compound named Westerdijkin A, with anticancer activity against K562 and HL-60 cell lines, was isolated from *Aspergillus westerdijkiae* in deep-sea sediments of the South China Sea, at a depth of 4593 m [109]. Acaromyester A and Acaromycin A are other cytotoxic compounds found in *Acaromyces ingoldii* FS121 from the depth of 3415 m in the South China Sea. These bioactive metabolites presented cytotoxic activities against cancer cell lines such as breast cancer (cell line MCF-7) and hypotriploid lung cancer (cell line NCI-H46) [110,111,112].

*Penicillium* is a genus of ascomycete fungus widely distributed in deep-sea sediments and has received a lot of attention due to its important secondary metabolites. Most of the secondary metabolites of *Penicillium* have been reported to have anticancer properties [113,114]. Recent studies are revealing the diversity of fungi in the Arabian Sea marine environment, but despite such biodiversity, the biological potential of sea fungi from the region is rarely studied [30]. One of the fungal strains separated from marine sediment specimens was recognized as *Penicillium dipodomyicola* by ITS rDNA sequencing and identified as *Penicillium* sp. ArCSPf [30]. Due to its ability to tolerate high salt concentrations, low temperatures, and pressure changes, *Penicillium* sp. ArCSPf is one of the most prevalent and dominant species of the area. Studies on fungal diversity from the Arabian Sea continental slope showed that approximately 43% of fungal isolates belong to the genus *Penicillium* [30,115]. The results of current research show that piezophilic or barophilic fungi can be considered as rich sources of natural products with significant antibiotic and cytotoxic activity [30]. Brevione isolated from *Penicillium* spp., living in deepest part of the sea, has illustrated strong cytotoxic activity [93,96]. In addition, terpenoid derivatives with potential cytotoxicity are the other class of compounds, frequently found in *penicillium* and *Aspergillus* species of the deep-sea waters [105,116]. Some of the most interesting research on these fungi and their identified metabolites, with biological activities like anticancer properties, are summarized in Table 2.

## 4. Acidophilic and Alkaliphilic Fungi

Fungi normally grow at a slightly acidic pH of around 5.0–6.0. In contrast, acidophiles grow optimally at pH values ranging from 3–4, whereas the alkaliphiles can thrive the higher pH values of above 9 [123]. 

Emericellipsin A is an example compound isolated from *Emericellopsis alkaline* and *Streptomyces* SPP (alkalophilic fungi strains) and is presumed to be an effective novel antitumor substance with a significant cytotoxic effect against HepG_2_ and HeLa tumour cell lines [69,124]. 

Moreover, many peptaibols have been isolated from *Emericellopsis sp* such as Emericellipsin A–E, where in vitro tests displayed selective and suppressing cytotoxic activity toward tumour cells by activating calcium-mediated apoptosis against HepG_2_ and Hela cell lines [125]. Penicillium species of the surface waters of an acid mine produce two activators of signal transduction enzyme inhibition, including caspase-1 (Casp-1) and matrix metalloproteinase-3 (MMP-3) inhibitors [126]. Specific Casp-1 inhibitors may serve as new drug with highly potent cytotoxic activities [127,128,129,130]. The specific MMP inhibitors represented a novel therapeutic path to treat cancers by obstructing the activity of MMPs abused by tumour cells. There are reports on stabilizing the tumour progression effect of these inhibitors when applied as low-toxicity supplements for cytotoxic treatments [131,132]. 

Berkeley Pit Lake, located in Butte, Montana, is famous for its abandoned open-pit copper mine 540 m below the Earth’s surface, saturated with metal-sulphate-contaminated acidic water of over 140 billion L [133,134,135,136]. In recent years, several studies have focused on the metabolites from the microorganisms growing in this harsh environment [41]. A novel spiroketal compound named berkelic acid and two new hybrid polyketide–terpenoid compounds known as berkeleydione and berkeleytrione have been isolated recently from the Penicillium species of the lake. These metabolites, along with several other compounds such as berkeleyacetals A–C from an unknown species of Penicillium, were believed to have biological activities such as anticancer properties [126,133,136]. Berkelic acid was tested against 60 human cell lines displaying selective anticancer properties against ovarian cancer OVCAR-3 with a GI_50_ of 9.13 (91 nM concentration) [133]. The chemical structure of berkelic acid was revised by the Fürstner group using methods like synthetic NMR and crystallographic techniques in 2018 [137]. *Penicillium rubrum* produces Berkeleyamides A–D and berkeleyones A–C in the low micromolar range that were tested positive for the suppression of MMP-3 and Casp-1 via interleukin 1-β inhibition in THP-1 cells [99,138,139]. *Penicillium solitum* and *Penicillium purpurogenum* JS03-21 produce a new tricarboxylic acid derivative and two new drimane sesquiterpene lactones named berkedrimanes A and B, which, at low micromolar concentrations, reduce the production of cytokines interleukin (IL-1β) by inflammasomes [128,140,141]. Berkazaphilones A and B, berkedienolactone, octadienoic acid derivatives, berkedienoic acid [100,101], vermistatin, dihydrovermistatin, penisimplicissin, aldehyde, azaphilone, and methylparaconic acid were isolated from a culture broth of *Penicillium rubrum* [134,139,142]. The compounds were isolated either for their inhibitory effect on the signal transduction enzyme Casp-1 or because of their structural similarity to such inhibitors [139]. Selected compounds were further evaluated for their ability to inhibit interleukin-1β production by inflammasomes in induced THP-1 cells. Berkazaphilones B and C and a penisimplicissin analogue of vermistatin showed selective activity against human leukaemia cancer cell lines [134,139] (Table 3).

## 5. Halophiles and Other Poly Extremophiles

Halophiles are organisms found in all domains of life, including bacteria, archaea, and eukarya, and are generally referred to as “salt-loving” organisms. The halophile fungi groups have a variety of properties and valuable secondary metabolites.

Genomic and molecular studies have revealed the differential expression of many genes involved in the biosynthesis of secondary metabolites in these organisms compared to their expression under normal conditions [151,152]. The obtained compounds from these fungi can potentially be used in industrial and biotechnological fields, as well as in the production of β-carotene, ectoine, and bio-rhodopsin for optical computing, and in holograms, biosensors, photoelectric devices, cosmetics, preservatives, fermented food products, the manufacturing of bioplastics and artificial retinas, bio-surfactants and exopolysaccharides, biofuels, pigments for colouring, and even bioremediation [153,154,155,156,157].

Halophiles are confined and adapted to environments where salt is present and, in many cases, is needed for their survival. In addition to the high salt concentrations, halophilic microorganisms are exposed to several types of other abnormalities, such as nutritional deficiencies, osmotic pH, UV, and ionic stress. Unlike halophilics, the non-tolerant organisms with no adaptability to these conditions lose water through their cells and tissues faster, during which the turgor pressure loss causes cytosolic dehydration [157,158,159]. Halophiles are considered one of the most valuable resources of bioactive compounds due to their low water activity that triggers the formation of rare secondary metabolites with potential anticancer properties [160,161]. Studies have shown that the biosynthesis of anticancer compounds such as cytochalasin E, ergosterol, and rosellichalasin from *Aspergillus* sp. increased at higher salt concentration of the medium [37,83]. The mentioned compounds diminished the durability of BEL-7402, RKO, A549, and Hela human cancer cell lines. Among them, ergosterol had the greatest inhibitory effect on a human colon cancer cell line [160,161,162]. The temperate and subtropical regions host *P. chrysogenum* or *P. rubens*, the producers of two interesting compounds named chloctanspirone A and B [135,163]. These compounds are the first chlorinated sorbicillinoids obtained from natural origins and are distinguished by their unique ring structures [37]. Chloctanspirone A is the more active analogue with a greater prohibitory effect on human leukaemia HL-60 and lung cancer cell line A-549 compared to chloctanspirone B, with its moderate or non-existent activity against the same cell lines [164]. 

The pullulan polymer molecules that have been produced by *A. pullulans* are considered important polysaccharides with biological activities [165,166]. Studies have demonstrated pullulan’s effect on the T and B cells by means of altering the immune-stimulatory system [167]. Pullulan treatment upregulates the costimulatory molecular expression and enhances the pro-inflammatory cytokine production in bone marrow-derived dendritic cells (BMDCs) in vitro and in spleen DCs in vivo [167,168]. Pullulan induced the maturation of DCs in spleen- and tumour-draining lymph nodes (drLN) and in tumour-bearing mice and promoted the OVA-specific T cell activation and migration of the T cells into the tumour [169,170]. The combination of OVA and pullulan inhibited tumour growth and liver metastasis [171,172]. It was demonstrated that treatment with a combination of pullulan and tyrosinase-related protein 2 (TRP2) peptide suppressed the B16 melanoma growth [173]. It was also found that pullulan can not only enhance the DCs’ maturation and function, but also it acted as an adjuvant in promoting antigen (Ag)-specific immunity in mice [166]. Thus, pullulan could be a new and useful adjuvant to be considered in therapeutic cancer vaccines. Another relative compound is liamocin oil, which is structurally unique and heavier than water, produced by certain strains of fungi. One of the highest yields for liamocin production (7.0–8.6 g/L) was reported by Zhang et al. 2016 [166] from RSU 9 and RSU 21 strains of *A. pullulans* originated from the tropical environment of Thailand. The liamocins prohibited a human cervical cancer cell line and two human breast cancer cell lines with IC_50_ values of 32.2 ± 1.4–63.1 ± 2.4 μg liamocins/mL with no adverse effects on healthy cell lines [168]. This suggests that these compounds deserve more screening as potential anticancer drugs with critical and appropriate clinical trials and precise molecular identifications [174,175]. Some halophiles and other extremophiles and their anticancer biomolecules are shown in Table 4.

**Figure 1:**
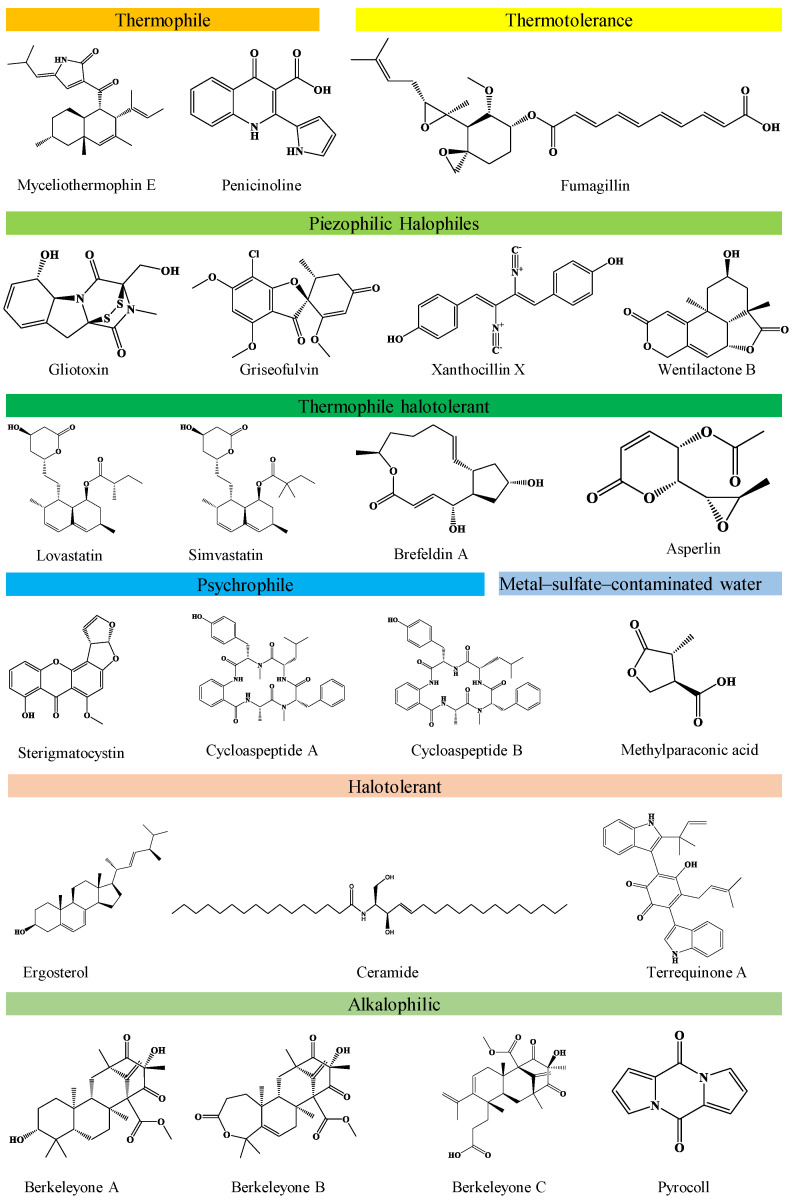
Chemical structure of the selected novel cytotoxic compounds from extremophilic fungi.

Fungi produce several pigments such as azaphilones, melanin, carotenoids, polyketides, etc., with positive roles in human well-being, while containing other harmful pigments. *A. pullulans* produces important pigments conferring strong anticancer activities with low cytotoxicity towards normal cells [166]. *Alternaria* sp. ZJ9-6B was screened for potential production of pigments against human breast cancer cell lines with positive outcomes. Pigments derived from *Monascus purpureus* like monascin presented prohibitory activity against mice skin carcinogenesis, while ankaflavin showed activity against Hep G2 and A549 human cancer cell lines [183,184]. Similarly, monaphilone A and B, isolated from *M. purpureus*, showed promising anti-proliferative effects on HEp-2 human laryngeal carcinoma cell lines [185,186,187]. Table 5 summarizes the other contaminated environments’ fungal metabolites with anti-cancer properties. The chemical structure of the selected novel antitumour compounds from different groups of extremophilic fungi is presented in Figure 1.

## 6. “Omics” Tools for Discovery of Bioactive Metabolites of Extremophilic Fungi

The biosynthetic pathways of bioactive metabolites of microorganisms in extreme environments can be revealed by the application of “Omics techniques” with better resolution and predictability to accelerate the generation of information for the natural products research communities. This approach has already been applied for studying single microorganisms or microbial communities in extreme environments [192,193,194]. In this section, an important algorithmic perspective behind several computing platforms, software, and databases is provided to provide an overview of the various computations for omics tools approaches to the elucidation of metabolic pathways of fungal metabolites in harsh environments. 

Some of the important fungi-related platforms are ‘antiSMASH’ ‘fungiSMASH’, ‘NP.searcher’, ‘ClustScan’, ‘CLUSEAN’, ‘SMURF, SBSPKS, ‘MIDDAS-M’, ‘CLUster’ ‘CASSIS/SMIPS’, ‘NRPSPredictor’, ‘SBSPKS’, ‘NaPDoS’, ‘GNP/PRISM’, ‘EvoMining’, and ‘C-Hunter’ [195,196,197]. Most of these tools are freely accessible to users with limited programming skills. They usually contain algorithms that can align and search for interactive regions at the level of gene clusters and find and compare similarities with their closest relatives from other databases. This is then followed by identification prediction of metabolites that are likely to be synthesized from the gene sequences that are already available in the databases. Extraction algorithms and scalable expression platforms give very wide access to fungus-derived secondary metabolites’ biosynthetic pathways [198,199]. These are promising tools for identifying the secondary metabolites and their biosynthetic gene clusters, evaluating the genetic potential of different strains, and more effectively discovering currently unknown metabolites [195,196]. In addition, this information can be considered for the metabolic engineering of molecules by using synthetic biology approaches [192,197]. 

Metabolic gene clusters include a group of co-located genes that encode enzymes for the biosynthesis of a particular metabolite and are prominent features of chromosomes; therefore, the sequencing of these known gene families provides information that can be used to understand the presence of a potential secondary metabolite biosynthesis pathway in different species [198]. There are two main strategies for exploiting bioinformatics tools to identify gene clusters that are involved in biosynthetic pathways with high accuracy [199]. The first step of the gene mining process is to identify genes encoding conserved enzymes/protein domains that have associated roles in secondary metabolism [200,201], such as “adenylation”, “condensation”, glycosylation, or peptidyl carrier proteins, and domains of nonribosomal peptide synthetases. In the second step, defined algorithms are specifically used to associate the presence of such similarities with similar classes of natural products for intra- or inter-species matches [192,202]. 

Bioinformatics methods are mainly used to primarily locate the main so-called “backbone” enzymes, such as nonribosomal peptide synthetases, polyketide synthases, and their hybrids (peptide synthetases-polyketide synthases), in order to accurately identify gene clusters from known families and very specific classes [203]. 

Meanwhile, modern algorithms have been successfully designed for identifying potential gene clusters using the de novo-based method, i.e., without prior knowledge of functional domains or known patterns of key enzymes. It should be noted that gene clusters for many of the specific metabolic pathways have been discovered in common filamentous bacteria and fungi, but more are now being found in plants and higher organisms [204]. 

Next-generation sequencing provides more and more opportunities for studying microbial diversity and for predicting rare metabolic pathways and genes that are involved in stress tolerance and the survival of microorganisms in extreme environments [205]. 

**Figure 2:**
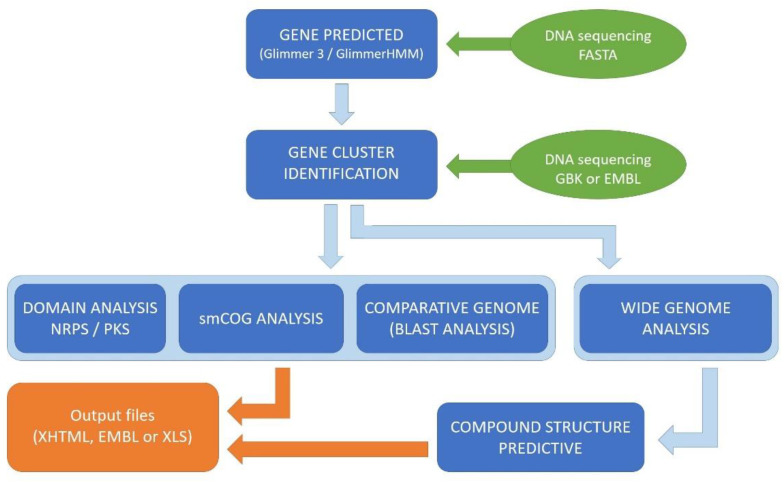
Steps of antiSMASH and SMURF algorithms’ analysis, from the input (sequenced DNA) to output files.

One of the key shortages in the applicability of “Meta-Omics” is the absence of real interactivity when new results for the new bioactive compounds are found. In this regard, studies based on genomic data can widely explain the genetic basis of secondary metabolite biosynthesis, particularly for the genes encoding for characteristic secondary metabolites in a better organized manner [206]. Additionally, genomics has opened a new door for the engineering of new analogues of many complex structural metabolites by sequencing the genomes of fungal species and identifying biosynthetic pathways [207]. The ability of genome mining to search for the biosynthetic roots of secondary metabolites based on the distribution of polyketide synthases and non-ribosomal peptide synthetase gene clusters can reveal previously undefined pathways. Therefore, the integration of genome mining, computational techniques, and analytical biochemistry could be the future of natural product research [208,209] (Figure 2). 

On the other hand, metabolomics and analytical chemistry techniques provide distinct strategies for metabolic engineering, which are provided by the hierarchical structure of the regulation of secondary metabolites’ biosynthesis, such as the manipulation of universal regulators to enhance the generation of secondary metabolites [210], or by targeting metabolic pathways for specific regulators to increase the content of a particularly interesting compound [208,211]. Culture condition screening is considered a successful and well-stablished experimental approach to detecting specialized secondary metabolites. In this method, organisms are exposed to different growth conditions or external stressors; then, the culture medium is analysed for secreted molecules using techniques such as mass spectrometry and nuclear magnetic resonance spectroscopy [212]. 

Developing modern computational techniques such as spectrum search algorithms, DEREPLICATOR+ is useful for identifying natural polypeptide products which contain ribosomal synthesized, or post-translationally modified, peptides and non-ribosomal peptide synthetase [213]. Machine learning can be extended for the identification of terpenes, benzenoids, flavonoids, polyketides, alkaloids, and other classes of natural products [214]. “DEREPLICATOR+” is one of the services that enable mutual validation of peptidogenomics/glycogenomics as a new genome mining strategy [214]. 

It should be emphasized that obtaining pure active compounds is a common problem in laboratories with limited facilities as it is accompanied by the cost and time of data reproduction and analytics. In this regard, it is important to consider the management of big data with proper analysis that can accelerate the bio-discovery efforts by providing rationale data for libraries and databases [215,216].

MeFSAT is an in silico chemical library with its primary objective being building a non-redundant resource of secondary metabolites of medicinal fungi along with information on their two-dimensional (2D) and three-dimensional (3D) chemical structures [217]. The information compiled in the MeFSAT database is openly accessible [218].

Advanced high-throughput sequencing technologies revolutionized microbiology, particularly in studies conducted in extreme environments, showing the high diversity and complexity of those microbial ecosystems. However, understanding the physiology of microorganisms in a particular environment, such as natural extreme environments or in vitro laboratory simulations, is now considered essential for completing genomic or transcriptomic studies that cannot be replaced by any other method. 

As a result, a combination of traditional cultivation methods and modern culture-independent techniques can be considered the best ways to better understand the growth and multiplication of microorganisms in harsh environments [219,220,221]. In several cases, the identification of novel metabolites originates from initial screening and is not researched with further genome sequencing and dereplication [222,223].

So, to summarize the above, with the help of approaches such as “Omics” and genome mining, optical measurement, real screening, and three-dimensional models, with the support of informatics-based analysis and artificial intelligence (AI), the dosage of the effective substance in a natural product can be determined for diagnosis of cancerous conditions and its phases [224,225,226]. Today, recent advances in “Omics” techniques can be utilized as tools for detecting cellular malfunctions that are at the centre of multi-factorial diseases such as cancer [227,228]. Genomics and proteomics are thought to enable the rapid [229], complete, and parallel analysis (pipeline processes) of genes and proteins that are expressed in a particular cell or type of tissue [230]. On the other hand, turning a normal cell into a cancer stage requires molecular changes at various levels, including the genome, epigenome, transcription, proteome, and metabolome [231]. In the field of cancer diagnosis, a variety of different profiles of cancer lines and natural tissues can be used to determine genomic or proteomic characteristics [232]. Since the availability of several genomes data and “Gene and protein expression profiles”, such data have had a significant impact on cancer research and therapies and led to genomics turning into a distinguished technique that has developed continuously by using genetic sequences in cancer research [233,234]. This method extensively qualifies the rate and probability of somatic mutations in individual patients and by applying big data to identify environmental factors, which are related to cancer, mutagenesis, and germline predispositions [235,236]. “Gene and protein expression profiles” also have the potential to improve the clinical management of cancers by providing classification, either by developing class prediction or class discovery or by providing information for diagnostic classification, and to identify new molecular targets to improve patient care through the identification of profiles that predict responsiveness to therapy or prognosis [237,238,239]. 

Finally, the generation of such information and turning it into theories depends on genome sequencing to create information systems to assist physicians in making decisions about the diagnosis and treatment of cancer in patients [239]. 

**Figure 3:**
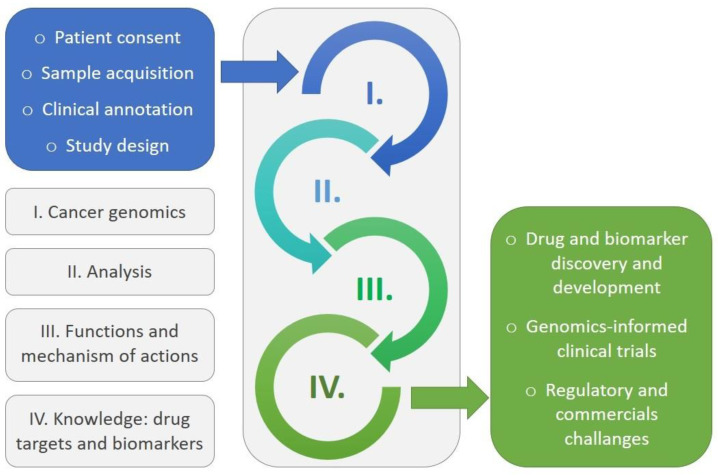
The stages of early clinical trials leading to cancer drug discovery.

A schematic view of the stages of early clinical trials leading to the discovery of cancer drugs is shown in Figure 3. The current and future of drug discovery can rely on the utilization of a combination of multi-omics and molecular and analytical approaches [240]. 

A collection of bioinformatics tools and databases for the identification of metabolic gene clusters related to drug discovery of extremophiles and extremotolerant fungi are summarized in Table 6. The details of the platform or tool, system requirements, input formats, function, output, and finally the accessibility options are included in this table.

A schematic view of the stages of early clinical trials leading to the discovery of cancer drugs is shown in Figure 3. In addition, we would like to address a new approach, which would be the chemical modification of the natural metabolites with conjugational alterations in laboratory and to investigate the potential functionality enhancement of such hybrid molecules [280,281].

## 7. Conclusions

In the fascinating world of microbiology, fungi that can survive and multiply in harsh environments have an unusual biochemistry that is highly regarded in biotechnology applications in industry, pharmaceuticals, environmental protection, medicine, and many other fields. Secondary metabolites of fungi from extreme environments are considered novel bioactive compounds that have attracted much attention in recent years. They produce essential, beneficial natural metabolites related to a variety of applications, including novel drugs and medicines to be applied in therapies as immunosuppressants, cytotoxic and anticancer agents, and antimicrobials, anti-inflammatories, and antioxidants. They also serve as resources for industrial and biotechnological applications as enzymes and nutrients, such as β-carotene, ectoine, and bio-rhodopsin for optical data processing; as bio-surfactants and exopolysaccharides in bioremediation and biofuels; and as pigments for the dye and food industries.

In the era of omics, on one hand, with the rapid development of recent tools and techniques, the alteration in culture conditions or the generation of mutants could also give rise to modified strains with the ability to produce novel important metabolites; on the other hand, these new methods can be used to identify valuable biological resources. Synthesizing such information and turning it into theories can take over the present and future of drug discovery as being referred to a combination of multi-omics and molecular and analytical approaches.

In this article, some of the most well-known and practical bioinformatics tools/platforms and databases for the study of fungal metabolism are presented, especially for those living in extreme environments.

In addition, around 260 compounds were collected from previous reports. To address the new trend of looking for medicines of natural origin, several compounds from extremophilic, extremotolerant, and poly (extremophile and tolerant) fungi were summarized in different tables of this work. These include compounds from fungi that reproduce, grow, and develop in extreme temperature conditions, compounds belonging to piezophilic or barophilic (high pressure) groups, compounds selected from fungi that can live in acidic or alkaline environments, compounds found in environments with high salt concentrations and low water activity, and, finally, compounds that were discovered in other types of extreme environment. The metabolites mentioned in this review have strong or moderate bioactive properties and may have the potential to be used in future cancer drug discovery and therapies. 

## Figures and Tables

**Table 1:** Investigated antitumour properties and target cell lines of fungi that proliferate, grow, and develop under extreme temperature conditions.

Species	Type of Extreme Fungi	Compounds				References
		Living and Thriving Habitats	Metabolites with Antitumour Properties	Effective Dose	Examples of Tissues or Cells Affected by Metabolites	
*Penicillium* *algidum*	Psychrophile	Soil, Zackenberg, Greenland	Cyclic nitropeptide Psychrophilin A (nitropeptide) Psychrophilin D (nitropeptide)	10.1 μg/mL	Cytotoxicity against P388 murine leukaemia cell lines	[73,89,93]
			Cycloaspeptide A, B	˃12 μg/mL	Antitumour activity in general	
*Friedmanniomyces endolithicus*	Psychrophile	Antarcticrocky deserts	Melanin pigments	–	Cytotoxicity against HFB4 cell line	[94]
*Aspergillus* sp.	Psychrophile	Alga-derived, North Sea, Germany	Cyclotripeptide Sterigmatocystin Psychrophilin E–F	4.4–28.5 μM	Antitumour activity in general Cytotoxicity against HCT-8 (colorectal), BEL-7402, BGC-823, A-549, and A–2780, HCT116 cell line	[73,95]
*Aspergillus clavatus C2WU*	Thermophile	Hydrothermal vents crab Kueishantao, Taiwan	Clavatustide A, B	15 μg/mL	HCC cell lines (Bel-7402, HepG2, and SMMC-7721), suppress HHC cell proliferation by inducing G1 arrest and suppressing the G1/S transition in a dose-dependent manner	[96]
*Mucor strictus*	Psychrophile	Shandong, China	Ergosterol	37–78 μM	HeLa and MCF-7 cell lines	[97]
*Myceliophthora thermophila*	Thermophile	Fumaroles soil, Taipei, Taiwan	Myceliothermophins A–E	0.26–1.05 μg/mL	A549, Hep3B, MCF-7, and HepG2cell lines	[26,98]
*Malbranchea sulfurea*	Thermophile	Hot spring, Taiwan	Malbranpyrroles A–F	3–11 µM	Stop G0/G1 phase in all MCF-7 and HepG2 cells cancer cell lines	[99]
*Aspergillus terreus*	Thermophile halotolerant	Fumaroles soil, Hot spring, Taiwan	Terrein	0.8 ± 0.3 μg/m	Suppressed the activity of ABCG2 transporters, activating the caspase-7 signalling pathway, and induced apoptosis by inhibiting the Akt pathway	[37,42,92,100]
			L–asparaginase	3.79–12.6 µg/mL	Cytotoxicity against HCT-116, Hep-G2, and MCF-7 cell lines	
			Lovastatin	0.6–15.6 μM	Cytotoxicity against lung carcinomas (DLRP, H1299), breast carcinomas (MCF-7, SKBR-3, BT474A, MDA-MB-453), and malignant melanomas (HT144, SK-MEL-28, M14)	
			Asperlin	1.1–110 nM	Inhibits breast cancer by induction of apoptosis and MCF-7; cytotoxicity against MCF-7 cell line	
*Thelebolus* sp. *IITKGP-BT12*	Psychrophilic Halophilic	Sabinar saline wetland in Spain	Exopolysaccharide (EPS)	275.42 μg/mL	Anti-proliferative activity against B16–F0 cells, with mediated apoptosis	[75,101]
*Oidiodendron truncatum* GW3–13	Psychrophile	Soil, Great Wall station (Chinese Antarctic station), Antarctica	Piperazines	0.3–28 nM	Antitumour activity in general	[77,102]
			Oidioperazines A–D (Epipolythiodioxopiperazines)	>10.1 μg/mL	Cytotoxicity against HCT-8, BEL-7402, BGC-823 (gastric), A-549, and A-2780 cell lines	
			Chetracins B–C (Epipolythiodioxopiperazines)	0.003–0.028 μg/mL		
			Chetracin D (Diketopiperazines)	0.14 ± 0.03–1.83 ± 0.07 μg/mL		
			Melinacidin IV, T988,T988 A	3–28 nM	Cytotoxicity against HCT-8, BEL-7402 -BGC-823 (gastric), A-549, and A-2780	
*Penicillium* sp. PR19 N–1	Psychrophile	Marine sludge, Prydz Bay, Antarctica	Eremophilane–type sesquiterpenes	5.2–85.8 μM	Cytotoxicity against HL-60 and A-549 cell lines	[30,42]
			Chloro–eremophilane sesquiterpenes	11.8–12.2 μM		
*Aspergillus* sp.	Psychrophile	Alga-derived, North Sea, Germany	Cyclotripeptide Sterigmatocystin	4.4–28.5 μM	Antitumour activity in general	[42]
*Trichoderma velutinum*	Psychrophile	Soil, Himalayan, India	Lipopeptaibols	2–7 μM	Antitumour activity in general	[42]
*Penicillium rubrum*	Thermophile/ acidiophile	Damp, water-damaged buildings or potato dextrose broth (pH 2.7)	Berkazaphilones A–C	10–100 μg/mL	Solid tumour cancers and activation of caspase-1 in prostate cancer and ovarian cancer	[103]
			Octadienoic acid derivatives			
			Berkedienoic acid			
			Berkedienolactone			
			Azaphilone			
			Vermistatin			
			Dihydrovermistatin			
			Penisimplicissin			
			Aldehyde			
			Methyl paraconic acid			
*Penicillium* sp.	Thermophile	Ghamiqa hot springs, Saudi Arabia (temperature range from 45°C to 65 °C)	3-(furan 12-carboxylic acid)-6-(methoxycarbonyl)-4-hydroxy-4-methyl-4	–	Cytotoxicity against the lymphoma human cancer cell line HTB-176	[37,42,90,103,104]
			5-dihydro-2H-pyran 1 3α-methyl-7-hydroxy-5-carboxylic acid methyl ester-1-indanone 2			
	Piezophilic Halophiles	Deep-water sediment sample that was collected at a depth of 5115 m in the East Pacific	Ethyl Acetate (Etoac)	2.6 µM	Inhibitor of acute myeloid leukaemia (AML) cells	
			Austinol	2–7.6 μM	Cytotoxicity against HTB-176 cell line	
			Emodin (1,3,8-trihydroxy-6-methylanthraquinone)			
			2-methyl-penicinoline 5	11.3 µM	Inhibitor of HepG2 cell lines	
			Penicinoline (γ-pyridone alkaloids)	11.8–12.2 µM		
			Methylpenicinoline γ-pyridone alkaloids	11.3–13.2 µM		
			Mycophenolic acid	0.5–1 μM	Inhibitor of human inosine 5′-monophosphate dehydrogenase (IMPDH)	
			Fudecadione A–B	12.6–24 μM	Cytotoxicity against NCI-H187, MCF-7, and KB cell lines	
			Ligerin	117 nM	Inhibitor of POS1 cell lines	
*Aspergillus fumigatus*	Thermotolerant	Soil and decaying organic matter, Conidia surviving at 70 °C	Fumagillin	8–40 µM	Inhibitor of B16, F10, A549, and L132 Cell line	[91]
			Gliotoxin	0.54–2.7 µM		
*Penicillium chrysogenum*	Thermotolerant	Damp or water-damaged buildings	Chloctanspirone A	9.2, 39.7 μM	Cytotoxicity against HL-60 and A-549 cell lines	[37]

**Table 2:** Investigated antitumour properties and target cell lines of piezophilic or barophilic (high pressures) fungi metabolites.

Species	Type of Extreme Fungi	Compounds				References
		Living and Thriving Habitats	Name of Metabolites with Antitumour Properties	Effective Dose	Examples of Tissues or Cells Affected by Metabolites	
*Dichotomomyces cejpii* F31-1	Piezophilic Halophiles	Hainan Sanya National Coral Reef Reserve, China.	Dichotomocej A–D	35–39.1 μM	Cytotoxicity against rhabdomyosarcoma (RD) and HCT116 cell line	[42,117,118,119]
*Penicillium* sp. SCSIO Ind16F01	Piezophilic Halophiles	Deep water, deep-sea sediment, Indian Ocean	Chromone (Epiremisporine B–C)	15.8–16.6 μM	Cytotoxicity against SGC7901, MCF-7, and K562 cell lines	
*Penicillium purpurogenum* G59	Piezophilic Halophiles	Deep water, deep-sea sediment Indian Ocean	Chromone (Epiremisporine B, B1), Chromone (Isoconiochaetone C), Remisporine B, Coniochaetone A, Methyl 8-hydroxy-6-methyl-9-oxo-9H-xanthene-1-carboxylate	54.7–144.3 μM	Cytotoxicity against K562, HL-60, HeLa, and BGC-823 cancer cell lines at 100 μg/mL	
*Aspergillus* sp. SCSIO Ind09F01	Piezophilic Halophiles	Deep-sea China	Diketopiperazine	0.015–95.4 μM	Cytotoxicity against H460, SF–268, MCF-7, HT-29, and CHO-K1 cell lines	[107]
*Penicillium* sp. F23-2	Piezophilic Halophiles	Deep-sea sediment (depth 5080 m)	Meleagrins D–E	5–<100–200 μg/mL	Cytotoxicity and Pro-apoptoticagainst A-549, HL-60, BEL-7402, and MOLT-4 cell lines	[42,120]
*Penicillium* sp.	Piezophilic Halophiles	Deep-sea sediment, Arabian Sea	Conidiogenones B–G (diterpenes)	0.038–6.7 μM	Cytotoxicity against A-549, A-549, and HL-60 cell lines	[42,116,121,122]
			Roquefortine groups (C, F–G), Xanthocillin X, Chrysogine	1.8–6.7 μM		
			Brevione I–H	7.44–28.4 μM	Cytotoxicity against HL-60, A-549, MCF-7 and HEP3B cell lines	
			Peniphenones A	5.9 to 9.3 μg/mL		
			Peniphenones D			
		Deep-sea sediment (depth 5115 m)	Breviane spiroditerpenoids Breviones I–K	7.44–32.5 µM	Cytotoxicity against MCF-7 and A549 cell lines	
*Aspergillus dimorphicus* SD317	Piezophilic Halophiles	South China Sea sediment, (depth 2038 m)	Wentilactone A–B	Low micromolar dose	Cytotoxicity against HGC-27, HeLa, A549 and HT29 cell lines	[93]
*Aspergillus versicolor*	Piezophilic Halophiles	Deep-sea sediment, Bohai Sea, China	Brevianamide	8.63 µM	Antitumour activity in general	[93,95]
		Pacific Ocean, China (depth 2326 m)	Emodin (2-(dimethoxymethyl)-1-hydroxyanthracene-9,10-dione)	3.9–62 μg	Moderate inhibitory activity against MRSA-CGMCC 1.12409 and MRSA ATCC-43300MIC	
*Penicillium brevicompactum* DFFSCS025	Piezophilic Halophiles	Deep-sea sediment, Hainan, China	Mycochromenic acid, Gliotoxin	5.6–13.71 μM	Cytotoxicity against HCT116 cell lines	[42,104]
*Aspergillus SCSIO* Ind09F01	Piezophilic Halophiles	Deep-sea sediment	Asperethers A	0.015–95.4 μM and < 0.03–0.894 μM	Cytotoxicity against A-549, K562, and Huh-7 cell lines	[42]
*Aspergillus wentii* SD–310	Piezophilic Halophiles	Deep-sea South China Sea (depth 2038 m)	Asperethers A, E	Micromolar range	Antitumour activity in general	[109]
*Aspergillus westerdijkiae* SCSIO 05233	Piezophilic Halophiles	Deep-sea hypersaline sediments, South China Sea (Depth 4593 m)	Circumdatin F–G	2.5–11.3 μM	Cytotoxicity against K562, HL60, myelogenous, and promyelocytic leukaemia cell lines	[109]
*Acaromyces ingoldii* FS121	Piezophilic Halophiles	South China Sea (Depth 3415 m)	Naphtha- [2,3-b] Pyrandione analogue, Acaromyester A, Thiazole analogue	100 μM range	Cytotoxicity against MCF-7, NCI-H460, SF-268, and HepG-2	[110,112]
*Penicillium brevicompactum* DFFSCS025	Piezophilic Halophiles	South China Sea deep-sea sediment	Brevianamide	15.6 μM	Cytotoxicity against HCT116 cell lines	[93]

**Table 3:** Investigated Antitumour properties and target cell lines of acidophilic or alkalophilic fungi metabolites.

Species	Type of Extreme Fungi	Compounds				References
		Living and Thriving Habitats	Name of Metabolites with Antitumour Properties	Effective Dose	Examples of Tissues or Cells Affected by Metabolites	
*Pithomyces* sp.	Acidophiles, Piezophiles and metal tolerance	Surface and deep-sea water (a 1500 ft deep) Berkeley Pit Lake with high pH (2.7), North America	Aromatic compounds (not named)	0.4–4.61 μg/mL	Cytotoxicity against A549, SK-OV-3, SK-MEL-2, XF-498, HCT-15	[143]
*Penicillium rubrum*	Acidophiles	Surface water in Berkeley Pit Lake, North America	Berkeleyones A–D	2.7–37.8 μM	Cytotoxicity against interleukin 1-β production and caspase-1 enzyme signalling in THP-1 cell lines	[65,138,139,142]
			Meroterpenes	0.37–0.64 µM	Cytotoxicity against MMP-3, NCI-H460, IL-1β RPMI-8226 cell, RPMI-8226, SR, RPMI-8226 cell lines as caspase-1 inhibitors	
			Berkedienolactone	10–100 μg/mL		
			‘Octadienoic acid (berkedienoic derivatives)	5.67 μM		
			Sesquiterpene	300 nM–30 μM	Moderate caspase-1-inhibiting activity and strong cytotoxicity against MMP-3 activity	
			Vermistatin	2.0–200 μg/mL	Inhibitors of caspase-1, production of IL-1β in THP-1 and leukaemia cell lines	
			Penisimplicissin	10–100 μg/mL		
			Dihydrovermistatin	20 μg/mL		
			Methylparaconic acid	–		
			Berkazaphilone B–C (Polyketoids)	2.0–20 μg/mL	Antitumour activity in general	
			Cysteine protease	0.0052–34 µg/mL	Suppressing the caspase-1 and MMP-3	
*Penicillium* sp.	Acidophiles	Surface water or algae-associated, in Berkeley Pit Lake, North America	Berkeleytrione (Hybrid–polyketideterpenoid)	6.40 μM	Suppressing the caspase-1 and matrix Metalloproteinase-3 (MMP-3), with moderate activity against lung cancer cell lines	[42,99,126,128,134,136,141,144,145,146]
			Sesquiterpene	300 nM–30 μM	Moderate caspase-1-inhibiting activity and strong activity against MMP-3 cell lines	
			Berkeleyacetal C, Drimane sesquiterpene, Berkelic acid Berkeleydione	Micromolar to millimolar range	MMP-3 and caspase-1 inhibitor activity in NCI–DTP 60 tumour cell lines; suppressing metalloproteinase-3 (MMP-3) and the cysteine protease caspase-1 (Casp-1), and cytotoxic activity against NCI-H460 cell lines	
*Aspergillus fumigatus* KMC901	Acidophiles	Contaminated acid mine Gangneung, South Korea	Diketopiperazine	0.24–0.9 μM	Cytotoxicity against DU145, AGS, A549, and HCT-116 cell lines	[147]
*Penicillium clavigerum*	Acidophiles	Water sample in Berkeley Pit Lake, North America	Phomopsolides A–F, Phomopsolide C	>100–150 μM	Suppressing MMP-3 and caspase-1	[134]
*Pleurostomophora* sp.	Acidophiles	Water sample, Berkeley Pit Lake, North America	Berkchaetoazaphilones B (Azaphilones)	1.1–10 μM	Inhibited the TNFα, IL-6, and IL-1β production in inflammation assay and significant cytotoxicity activity against Y79 and LOX-IMVI cell lines	[148]
*Penicillium solitum*	Acidophiles	Water sample, Berkeley Pit Lake, North America	Berkedrimanes A–B (Sesquiterpene lactones), Tricarboxylic acid, Berkeleyacetals C	Micromolar range	Suppressing caspase-1 and caspase-3 in the micromolar range and mitigated the production of IL-1β by intact inflammasomes at low micromolar concentrations	[42,134,149]
*Emericellopsis alkalina*	Alkali-tolerant or Alkalophilic	Alkaline soil samples of Zheltyr Lake, Russia	Emericellipsins A–E	2.30 ± 0.30–8.00 ± 1.04 μM	Cytotoxicity against HMO2, HCT-116, MCF 7, K-562, B16, MDA-MB-231, HepG2, etc.	[124,125]
*Streptomyces* sp. AK 409	Alkali-tolerant or Alkalophilic	Water samples of high Ph	Pyrocoll	0.65±0.09 μg/mL	Antitumor activity against HMO2, MCF 7, HepG2, etc.	[42,125,150]
			Emericellipsin A	2.8 and <0.5 μM	HepG2 and HeLa cell lines	

**Table 4:** Investigated Antitumour properties and target cell lines of halophilic and halotolerant fungi metabolites.

Species	Type of Extreme Fungi	Compounds				References
		Living and Thriving Habitats	Name of Metabolites with Antitumour Properties	Effective Dose	Examples of Tissues or Cells Affected by Metabolites	
*Aspergillus* sp.	Halotolerant	Sonoran Desert, Arizona	Terrequinone	0.6–1.2 mM	Cytotoxicity against MCF-7, SF-268, and NCI–H460 cell lines	[29,37,42,107,176]
			11-methoxycurvularin, 11-hydroxycurvularin, Dehydrocurvularin, Penicillic acid, 13-deoxy-12(13)-dehydroterrefuranone	1.1–2.5 μM	Cytotoxicity against MIA Pa Ca-2, MCF-7, SF-268, NCI–H460, and MIA Pa Ca-2 cell lines	
			Aspochalasins	0.6–1.2 mM	Antitumour activity in general	
			Ergosterol	3.3 μM	Selective activity against RKO cells	
			Cytochalasin E	37–78 μM	Cytotoxicity RKO, A-549, and BEL-7402, All A549, Hela, BEL-7402, and RKO cell lines.	
			Rosellichalasin	–		
*Aspergillus* sp. F1	Halotolerant	Solar saltern, Shandong, China	Aspochalasins A, C–D, TMC-169, Qunine	37–78 μM	Cytotoxicity against MCF-7, SF-268, and NCIH60 cell lines	[42]
*Aspergillus* *variecolor* B-17	Halophilic, Halotolerant, Xerophilic	Jilantai salt field, Inner Mongolia, China	Variecolortides A–C (Alkaloids)	61, 69, and 71 μM	Cytotoxicity against K-56 2 human leukaemia cell line	
			Indole-3-ethenamide	3.027 μM	Cytotoxicity against P388, A549, HL-60, and BEL-7402 cell activity against DPPH cell lines	[42,176,177]
			2-heptyl-3,6-dihydroxy-5-(3-methyl-2-butenyl) ben-zaldehyde and 2-(3E,5E-heptadienyl)-3,6-di-hydroxy-5-(3-methyl-2-bu-tenyl) benzaldehyde	9.99–203 μmol		
			Tetrahydroauroglaucin (As-pergin)	3–30.5 μM	Cytotoxicity against human U251 and glioma U87MG cell lines	
			Flavoglauci	–		
			Variecolorquinones A, B	1.3 and 3.7 μM	Antitumour activity in general	
*Myrothecium* sp. GS-1	Halophilic	Saline soil, Gansu, China	Ceramide(N-acetyl-3, 5, 11, 18-tetrahydroxyoctadecyl-2-amine)	63.6 μM	Cytotoxicity against PC-3, HL-60, and MCF-7 cell lines	[42,64,178]
			Variecolorins A–K	3.3 μM		
*Aspergillus versicolor* KR87	Halophilic	Soil fungi from the salt desert Little Rann of Kutch, India	Carnosol, Isouvaretin, Ginsenoside Rg5, Boesenbergin A	6.98–12.8 g/mL	Antitumour activity in general	[179]
			Tegafur	0.59–1.38 μg/mL		
			Procarbazine	0.15 mM		
*Penicillium chrysogenum* or *Penicillium rubens*	Halotolerant	Subtropical, salted, and damp or water-damaged buildings	Chloctanspirone A and B	9.2 and 39.7 μM	Cytotoxicity against HL-60 and lung cancer and A-549 cell lines	[122,163]
*Aspergillus flocculosus* PT05-1	Halotolerant	Putian saltern of Fujian Province of China	(22R,23S) -epoxy-3b,11a,14b,16b-tetrahydroxyergosta-5,7-dien-12–one, 6- (1H-pyrrol-2-yl) hexa-1,3,5-trienyl-4-methoxy-2H-pyran-2-one, Ergosteroid 1, 7-nor-ergosterolide, 3b-hydroxyergosta-8,24(28) -dien-7-one	12–18 μM	Cytotoxicity against HL-60 and BEL-7402 cell lines	[180]
*Aspergillus sclerotiorum* PT06-1	Halotolerant	Salt field, Fujian, China	Ceramide (N-acetyl-3, 5,11, 18-tetrahydroxyoctadecyl-2-amine)	63.6 μM	Cytotoxicity against PC-3, HL-60, and MCF-7 cell lines	[37,42]
			Asperphenamate	92.3–97.9 μM	Cytotoxicity against T47D, MDA-MB-231, and HL-60 cell lines	
*Aspergillus repens strain* K42	Xerophilic	Rice sample collected in Korea	Anthraquinones2-(dimethoxymethyl)-1-hydroxyanthracene-9,10-dione	3.9–62 μg/mL	Inhibitory activity against MRSA CGMCC 1.12409 and MRSA ATCC 43300	[42,181]
			Alkaloids			
*Chaetomium globosum*	Xerophilic Endophytic	Sonoran Desert, Arizona	Globosumones A–B, Orsellinic acid esters	3.4–9.5 μM	Cytotoxicity against SF-268, MCP-7, MIA Pa Ca-2, and NCI-H460 cell lines	[42,182]
*Aspergillus flavipes*	Xerophilic endophytic	Sonoran Desert, Arizona	Asperphenamate	3.0 and 18.3 μM	Moderate cytotoxic activity against several cancer cell lines	[42,183,184]
			Aspochalasins A, C–D, TMC-169	3.4–9.5 μM	Cytotoxicity against MCF-7, SF-268, and NCIH60 cell lines	
			Flavichalasines A–F	9.6–26.6 μM	Cytotoxicity against HL60, NB4, 231, HEP-3B, HCT116, and RKO cell lines	
*Cladosporium cladosporioides*	Xerophilic Psychrophilic	Saprotroph occurring as a secondary infection on decaying or necrotic parts of plants	Perylenequinones	1 μM/L	Induce apoptosis when Hce-8693 cells were incubated with photoactivated EA, HA, and HB	[42]
			Cladochromes F, G	0.05–50 μM	Antitumour activity in general	
*Monascus* sp.	Halotolerant Xerophilic Halotolerant	Non-sterile dried red fermented rice sample	Monaphilone A, B Ankaflavin Canthaxanthin Monascorubramin Rubropunctatin	55.3–77.6 μM	Cytotoxicity against Hep G2, Hep-2, and A549 human cancer cell lineS	[185,186,187]
*Aureobasidium pullulans*	Halotolerant	Salt farm soil	Pullulan	32.2–63.1 μg liamocins/mL	Cytotoxicity against liver cancer cells	[167,169,171]
			Soluble glucans	174.29 μg/mL		

**Table 5:** Investigated Antitumour properties and target cell lines of other contaminated environments’ fungal metabolites.

Species	Type of Extreme Fungi	Compounds				References
		Living and Thriving Habitats	Name of Metabolites with Antitumour Properties	Effective Dose	Examples of Tissues or Cells Affected by Metabolites	
*Penicillium simplicissimum*	Heavy metal-tolerant Cd (II)	Decaying vegetation	Dihydrovermistatin	8 µM	Plays a role in regulating inflammation and apoptosis. Inhibition of the signal transduction enzyme caspase-1, activity against 60 human cell lines	[188,189]
*Penicillium rubrum*	Metal-sulphate-contaminated water	Berkeley Pit Lake, a depth of 270 m abandoned open-pit copper mine Butte, Montana	Aldehyde	–	Signal transduction enzyme caspase-1 or because of their structural similarity to these inhibitors and ability to inhibit interleukin-1β production by inflammasomes in induced THP-1 cells	[42,99,104,134]
			Methylparaconic acid			
			Vermistatin analogue	0.28–33.9 µM		
			Penisimplicissin	0.33–0.66 mM		
		Acid mine waste lake in Montana	Berkazaphilones A–C	20 μg/mL	Cytotoxicity to leukaemia cancer cell lines through inhibition of caspase-1	
			Azaphilone-type polyketides		Exhibited potent cytotoxic effects against human retinoblastoma, leukaemia, and melanoma cell lines	
			Red pigment berkchaetorubramine	–		
*Alternaria* sp. ZJ9-6B	Extreme temperatures and UV-radiation tolerant	With Mangrove (*Aegiceras corniculatum*) populations South China Sea	Alterporriol K, M	13.1–29.1 μM	Cytotoxicity against MDA-MB-435 and MCF-7 cell lines	[42,190]
			Resveratrol			
			Porritoxin			
*Penicillium chrysogenum* or *Penicillium rubens*	Thermophile	Subtropical regions, damp or water-damaged buildings	Chloctanspirone A, B	9.2–39.7 μM	Cytotoxicity against leukaemia HL-60 and A-549 cell lines	[163]
*Alternaria tenuis* Sg17-1	Extreme temperature- and UV-radiation-tolerant	Damp or water-damaged buildings	Isocoumarins B, F	0.002–0.02 mM	Cytotoxicity against A375-S2 and Hela cell lines	[191]
			Sg17-1-4			
*Abortiporus biennis*	Cu_2_O- and/or Al_2_O_3_-tolerant	Roots or wood chips of deciduous trees, Asia, America, and Europe	Laccases	6.7, 12.5, 9.2 mM	Cytotoxicity against Hep G2 and MCF-7 cells and inhibitory activity against HIV-1 RT	[99,143]
			Isocoumarins B, F			
*Pithomyces* sp.	Acidophiles, piezophiles, and metal ions tolerance	Berkeley Pit, North America (depth of 1500 ft)	Aromatic compounds, Pitholides A–D	–	Cytotoxicity against brine shrimp cancer cell lines	[42,123]

**Table 6:** Computational, “Omics”, and bioinformatic tools and databases for identification of metabolic gene clusters related to drug discovery of extremophiles and extremotolerant fungi.

Name of Tools	System Requirements	Inputs	Functions, Outputs, and Accessibility Approaches	References
AntiSMASH and FungiSMASH’	- Web server: Any recent Web browser in any OS - Stand-alone version: Windows XP/ - Vista/7, Ubuntu Linux, or Mac OS	- List of GenBank/Ref Seq accession numbers - DNA sequences in GenBank/EMBL/FASTA formats - Bacterial and fungal sequences	- Interactive output with annotation and structure for each cluster - Graphical representation - Homologous clusters - BLAST/Pfam results - Functional predictions - Backbone genes and clusters - BGC analysis - Domain analysis - BGC database - Databases - Automated genome mining - https://antismash.secondarymetabolites.org/#!/start (accessed on 28 June 2023)	[38,148,241,242,243,244,245,246,247,248,249]
SMuRF	- Any Web browser in any OS - Open-source and freely available on GitHub - The method is implemented in R	- Takes multiFASTA, SNVs in BED, or vcf formats - Files that contain IDs in FASTA headers and sequences - Protein fungal sequences in FASTA format - Tab-separated files where data items are separated by the tab containing - Chromosomal coordinates of genes	- List of predicted backbone genes - Functional predictions for genes - List of predicted clusters - Backbone genes and clusters - Identification of significantly mutated regulatory elements - The only tools that can comprehensively detect complete secondary metabolite biosynthesis gene clusters - SMuRF predicts both SNVs and indels with high accuracy in genome or exome-level sequencing data. - Synthases (NRPS, PKS, DMATS, and NRPS–PKS) and coordinates of genes - https://github.com/skandlab/SMuRF (accessed on 28 June 2023)	[213,214,242,243,250,251,252]
CLUSEAN/Cluster Sequence Analyzer	- Preferred OS: Linux/Unix; except for NRPSPredictor - Open-source genome mining pipelines integrates standard analysis tools - Compatible with MS Win 2000, XP, and VISTA - Installation required: - –NCBI BLAST - HMMer - SVMlight - NRPSPredictor - Artemis - Perl 5.8 or higher	- EMBL-formatted DNA files - Bacterial and fungal sequences	- Domain content - Functional predictions for genes - Substrate specificity predictions - NRPS and PKS genes and protein domains - Bioperl-based annotation pipeline for secondary metabolite biosynthetic gene clusters. - CLUSEAN integrates standard analysis tools, like BLAST and HMMer, with specific tools for the identification of the functional domains and motifs in nonribosomal peptide synthetases (NRPS)/type I polyketide synthases (PKS) and the prediction of specificities of NRPS. - Results are annotated in EMBL files but can also be exported in MS Excel - Annotation and analysis of secondary metabolite gene clusters - https://bitbucket.org/tilmweber/clusean (accessed on 28 June 2023)	[120,244,247]
GNP/PRISM	- Any Web browser in any OS - Implementing: Chemistry Development Kit1 HMMER2 BLAST3 BioJava4 FIMO5	- Upload protein and DNA sequence file - Microbial genome sequence as input	- MassSpec guided peptidic natural products - Web application and algorithm to analyse and predict microbial genomes, biosynthetic gene clusters (BGCs), domain, PKS/NRPS, glycosylations, and to generate combinatorial libraries of structure predictions. - Provide bio- and chemoinformatic solutions for genomic natural product discovery. - NRPS, PKS, and NRPS–PKS, glycosylations’ structure prediction and dereplication - https://prism.adapsyn.com/ (accessed on 28 June 2023) - https://magarveylab.ca/ (accessed on 28 June 2023)	[74,248,253,254,255,256,257,258,259,260]
DEREPLICATOR+	- Any Web browser in any OS	- Mass spectrometry data	- Developed for the identification of known natural products from high-resolution - Database of chemical structures - Generates in silico mass spectra of compounds by predicting how they fragment during mass spectrometry. - Compares them to experimental LC/MS–MS and detects similarities - Identify polyketides, lipids, terpenes, benzenoids, alkaloids, flavonoids, aromatic, and aliphatic natural products. - https://cab.spbu.ru/software/dereplicator-plus/ (accessed on 28 June 2023)	[99,246,250,261,262,263,264,265,266,267,268,269]
SBSPKS	- Any Web browser in any OS	- Protein sequences in FASTA format (<10) - Sequences have to be entered manually by copying and pasting - Bacterial and fungal sequences	- Visualization of PKS domains - Prediction of docking order of PKSs - Prediction of PKS 3D structures - PKS genes; only domain analysis of BGC database - http://www.nii.ac.in/~pksdb/sbspks/master.html (accessed on 28 June 2023)	[214,254,255]
ClusterMine 360	- Any Web browser in any OS	- Sequence records in repository databases including: AntiSMASH Indigo Chem API ChemSpider NCBI	- Web accessible database of 297 BGCs - Database of microbial PKS/NRPS biosynthesis of other associated enzymes - They are also linked to the producing organism’s lineage - https://ngdc.cncb.ac.cn/databasecommons/database/id/356 (accessed on 28 June 2023)	[270]
IMG–ABC	- Any Web browser in any OS	- DNA sequences	- Web accessible database of 2489 BGCs, tightly integrated into JGI’s IMG platform. - https://img.jgi.doe.gov/cgi–bin/abc/main.cgi (accessed on 28 June 2023)	[271]
MIBiG	- Any Web browser in any OS	- DNA sequences	- A standard approved by the Genomic Data Standard and Repository - Consortium (GSC) - Defining a minimal set of information to describe BGCs - Developing the MIBiG standard - A huge number >500 BGCs have ben manually re-annotated by experts - Web accessible repository of 1393 BGCs - https://mibig.secondarymetabolites.org/ (accessed on 28 June 2023)	[272,273]
C–Hunter	- Any Web browser in any OS	- Uses GO and constructs DAGs	- Identifies Clusters Based On The Premise Of Shared Go Information - http://www.corehunter.org/ (accessed on 28 June 2023)	[274]
NRPS Predictor	- Any Web browser in any OS	- Protein sequences in FASTA format or - Extracted domain A signatures - Bacterial and fungal sequences	- List of domains - NRPS substrate and domain specificity - SVM scores for domains - NRPS and NRPS–PKS genes only - Predicts structures of secondary metabolites biosynthesized by type I modular PKS - Compares PRISM and GRAPE outputs for likelihood of backbone - Standalone application to correlate peptide sequence tags with NRP and RiPP BGCs - Prediction of modular PKS and domain specificity domain analysis - A domain specificity - https://bio.tools/NRPSpredictor2 (accessed on 28 June 2023)	[259,260,261,275]
ClustScan Professional	- Java Runtime Environment	- DNA sequences in FASTA or any format supported by ReadSeq - Bacterial and fungal sequences	- Annotated genes - Domain prediction - Browsing by domain searches - Backbone genes only - https://www.secondarymetabolites.org/mining/#clustscan-professional (accessed on 28 June 2023)	[254,255,256,257,258,276]
DECIPHER^Ⓡ^	- Any Web browser in any OS - R programming language	- Sequence databases: import, maintain, view, and export a massive number of sequences	- Sequence alignment: accurately align thousands of DNA, –RNA, or amino acid sequences; quickly find and align the syntenic regions of multiple genomes - Oligo design: test oligos in silico or create new primer and probe sequences optimized for a variety of objectives - Manipulate sequences: trim low-quality regions, correct frameshifts, reorient nucleotides, determine consensus, or digest with restriction enzymes - Analyse sequences: find chimeras, classify into a taxonomy, predict secondary structure, create phylogenetic trees, and reconstruct ancestral states - Gene finding: predict genes in a genome, extract them from the genome, and export them to a file - Search ngine and database - http://www2.decipher.codes/index.html (accessed on 28 June 2023)	[276]
MeFSAT	Any Web browser in any OS	- DNA sequences	- In silico chemical library, store their two-dimensional (2D) and three-dimensional (3D) chemical structure - https://cb.imsc.res.in/mefsat/ (accessed on 28 June 2023)	[277]
NaPDoS^®^	Any Web browser in any OS	- Sequences of all reference KS and C domains were aligned: MUSCLE ClustalX - BLOSUM 62	- Natural Product Domain Seeker - NaPDos is the rapid detection and analysis of secondary metabolite genes designed to detect and extract C– and KS–domains from DNA or amino acid sequence data, including PCR amplicon products, individual genes, whole genomes, and metagenomic data sets - Polyketide synthase (PKS) and nonribosomal peptide synthetase (NRPS) genes - NaPDoS analyses are based on the phylogenetic relationships of sequence tags derived from polyketide synthase (PKS) and nonribosomal peptide synthetase (NRPS) genes, respectively. The sequence tags correspond to PKS-derived ketosynthase domains and NRPS-derived condensation domains and are compared to an internal database of experimentally characterized biosynthetic genes - NaPDoS provides a rapid mechanism to extract and classify ketosynthase and condensation domains from PCR products, genomes, and metagenomic datasets. - Web application offering phylogenomic analysis of PKS–KS and NRPS–C domains - https://npdomainseeker.sdsc.edu/ (accessed on 28 June 2023)	[278,279]
NP.searcher	- Any Web browser in any OS	- DNA sequences in FASTA - Bacterial and fungal sequences	Coordinates for NRPS/PKS - Number of PKS/NRPS modules - Backbone genes only - Genome mining - Domain analysis - https://dna.sherman.lsi.umich.edu/ (accessed on 28 June 2023)	[164]
CASSIS/SMIPS	- Any Web browser in any OS	- Upload protein and DNA sequence file - InterProScan output file is required as input file for SMIPS	- A tool to predict secondary metabolite (SM) *anchor genes*, also called SM backbone genes, in protein sequences - Common SM anchor genes are PKS, NRP, and DMATS - The predictions are based on protein domain annotations made by InterProScan - Predict secondary metabolite (SM) gene clusters around a given anchor (or backbone) gene based on transcription factor binding sites shared by promoter sequences of the putative cluster genes. - https://sbi.hki–jena.de/smips/index.php (accessed on 28 June 2023)	[262,263,264]
MIDDAS-M	- Any Web browser in any OS	- Genome sequencing and transcriptome data	- Gene annotation, proteins and transcriptome data analyser - Motif-independent de novo detection of secondary metabolite gene clusters - https://www.secondarymetabolites.org/mining/#middas-m (accessed on 28 June 2023)	[262,265]
FunGeneClusterS	- Any Web browser in any OS R-based webserver and offline version available.	- DNA sequences	- Prediction of fungal gene clusters based on genome and transcriptome data - NRPS, PKS, DMATS and co-expression data BGC boundary prediction - https://www.secondarymetabolites.org/mining/#fungeneclusters (accessed on 28 June 2023)	[266,267]
SeMPI	- Any Web browser in any OS Uses antiSMASH and StreptomeDB 2.0 as backend engines	- DNA sequences	- Predicts structures of secondary metabolites biosynthesized by type I modular PKS - Considered as a dereplication tool. - Compares PRISM and GRAPE outputs for likelihood of backbone - https://www.secondarymetabolites.org/mining/#sempi (accessed on 28 June 2023)	[108,268]
GRAPE	- Any Web browser in any OS Works with PRISM	- Microbial genome sequence as input	- PKs and NRPs and NRPs–PKs retrobiosynthesis and dereplication - Alignment algorithm (GARLIC)	[250,255,268]
GARLIC	- Any Web browser in any OS	- Microbial genome sequence as input	- Retrobiosynthesis Pks And Nrps - Compares Prism and Grape outputs for likelihood of backbone - https://github.com/magarveylab/garlic-release (accessed on 28 June 2023) - https://ngdc.cncb.ac.cn/databasecommons/database/id/3320 (accessed on 28 June 2023)	[220,250]
SeMPI	- Any Web browser in any OS	- Microbial genome sequence as input	- Predicts structures of secondary metabolites biosynthesized by type I modular PKS - Compares PRISM and GRAPE outputs for likelihood of backbone - http://www.pharmaceutical–bioinformatics.de/sempi (accessed on 28 June 2023)	[268]
SEARCH-PKS/NRPS-PKS/SBSPKS	- Any Web browser in any OS	- Using An Assembly-Line Mechanism	- Identification of enzymatic domains in PKSs - Web application to mine for PKS BGC - A program for detection and analysis of polyketide synthase domains - SEARCHPKS (2003) is a software for detection and analysis of polyketide synthase (PKS) domains in a polypeptide sequence - Assessment of the sequence homology of various polyketide synthase domains - Identification of polyketide products made by PKS clusters found in newly sequenced genomes - https://www.secondarymetabolites.org/PKSNRPStools/#searchpksnrps-pkssbspks (accessed on 28 June 2023)	[192]
ClusterFinder	- Python (2.X)/ Numpy	Input File: Example_Input.Txt: Column Description include: - GeneID - Sequencing status - Organism name - Scaffold OID - Organism OID - Locus Tag - Gene Start - Gene End - Strand - Pfam Template Start - Pfam Template End - Pfam Start - Pfam End - PfamID - Pfam E–score - Enzyme ID	- Detect putative secondary metabolite gene clusters in genomic and metagenomic data; Clusterfinder (version: 1.0.1.)is available as standalone software and integrated into antiSMASH and IMG–ABC - Output [organims_name.out]: same as input + a column with probability values - OUTPUT2 [organism_name.clusters.out]: same as OUTPUT1, but only for the domains from gene clusters that have passed the filtering steps - https://github.com/petercim/ClusterFinder (accessed on 28 June 2023)	[16]
Pep2Path	- Any Web browser in any OS	- Multiple nucleotide sequence files (or files that contain multiple nucleotide sequences themselves) can be analysed at the same time. FASTA, GenBank EMBL formats	- An application to correlate peptide sequence tags with NRP and RiPP BGCs in bacteria and output chemical structure - Databases from local files - Accelerates the peptidogenomics method and facilitates a crucial step in the drug discovery pipeline - Identification of biosynthetic gene clusters for peptides analysed via tandem MS approaches - Consists of two main programs - Nrp2Path on nonribosomal peptides (NRPs) - RiPP2Path on ribosomally synthesized and post-translationally modified peptides (RiPPs) - http://pep2path.sourceforge.net/ (accessed on 28 June 2023)	[237,247,248]
GNP/PRISMR	- Any Web browser in any OS - implementing: Chemistry Development Kit1 HMMER2 BLAST3 BioJava4 FIMO5	- Microbial genome sequence as input	- Web application and algorithm to mine microbial genomes - Analysis of BGCs and domain, mainly PKS/NRPS, including glycosylations - Identification of biosynthetic gene clusters and generation of combinatorial libraries of structure predictions - Provide bio- and chemoinformatic solutions for genomic natural product discovery - Identification of biosynthetic gene clusters and generation of combinatorial libraries of structure predictions - https://prism.adapsyn.com/ (accessed on 28 June 2023)	[248,253,274]
EvoMiningN	- Any Web browser in any OS	- DNA sequences	- Web application for phylogenomic approach of cluster identification to mine for PKS BGCs - https://www.secondarymetabolites.org/mining/#evomining (accessed on 28 June 2023)	[274]
Java Treeview	- Any Web browser in any OS	Cross-platform rewrite extensions to the file format that allows the results of additional analysis	- Visualization of microarray data - Handles very large datasets well - http://jtreeview.sourceforge.net/ (accessed on 28 June 2023)	[279]

## Data Availability

Not applicable.
